# Adalimumab Monotherapy or Combination Therapy With Methotrexate in Paediatric Uveitis: Data From the AIDA Network Uveitis Registry

**DOI:** 10.1111/ceo.14534

**Published:** 2025-04-04

**Authors:** Carla Gaggiano, Alejandra de‐la‐Torre, Silvana Guerriero, Stefania Costi, Gaafar Ragab, Maria Pia Paroli, Emanuela Del Giudice, Luciana Breda, Saverio La Bella, Marco Cattalini, Maria Cristina Maggio, Alex Fonollosa, Ester Carreño, Maria Tarsia, Rosanna Dammacco, Soad Hashad, Antonio Vitale, Lampros Fotis, Stefano Gentileschi, Francesca Minoia, Germán Mejía‐Salgado, Halah Etayari, Paola Saboya‐Galindo, María Andrea Bernal‐Valencia, Adele Civino, Maria Sole Chimenti, Valeria Caggiano, Maria Francesca Gicchino, Jurgen Sota, Angela Mauro, Giuseppe Lopalco, Kalpana Babu Murthy, Mohamed Tharwat Hegazy, Vishali Gupta, Francesco La Torre, Rana Hussein Amin, Bruno Frediani, Gian Marco Tosi, Luca Cantarini, Claudia Fabiani, the AIDA Network

**Affiliations:** ^1^ Rheumatology Unit, Department of Medical Sciences, Surgery and Neuroscience University of Siena, Siena University Hospital [European Reference Network (ERN) for Rare Immunodeficiency, Autoinflammatory and Autoimmune Diseases (RITA) Center] Siena Italy; ^2^ Neuroscience Research Group (NEUROS), Neurovitae Center for Neuroscience, Institute of Translational Medicine (IMT), School of Medicine and Health Sciences Universidad del Rosario Bogotá Colombia; ^3^ Department of Ophthalmology and Otolaryngology University of Bari Bari Italy; ^4^ Unit of Pediatric Rheumatology, Azienda Socio‐Sanitaria Territoriale (ASST) Gaetano Pini‐Centro Specialistico Ortopedico Traumatologico (CTO) Milan Italy; ^5^ Internal Medicine Department, Rheumatology and Clinical Immunology Unit, Faculty of Medicine Cairo University Giza Egypt; ^6^ Faculty of Medicine Newgiza University (NGU) Giza Egypt; ^7^ Uveitis Unit, Department of Sense Organs, Eye Clinic Sapienza University of Rome Rome Italy; ^8^ Pediatric and Neonatology Unit, Department of Maternal Infantile and Urological Sciences Sapienza University of Rome, Polo Pontino Latina Italy; ^9^ Pediatric Rheumatology Unit S.S. Annunziata Hospital Chieti Italy; ^10^ Pediatric Clinic University of Brescia and Spedali Civili di Brescia [European Reference Network (ERN) for Rare Immunodeficiency, Autoinflammatory, and Autoimmune Diseases (RITA) Center] Brescia Italy; ^11^ University Department PROMISE “G. D'Alessandro” University of Palermo Palermo Italy; ^12^ Department of Ophthalmology Biocruces Bizkaia Health Research Institute, Cruces University Hospital, University of the Basque Country Barakaldo Bizkaia Spain; ^13^ Department of Retina Instituto Oftalmológico Bilbao Bilbao Spain; ^14^ Hospital Universitario Fundacion Jimenez Diaz Madrid Spain; ^15^ Clinical Pediatrics Siena University Hospital [European Reference Network (ERN) for Rare Immunodeficiency, Autoinflammatory and Autoimmune Diseases (RITA) Center] Siena Italy; ^16^ Tripoli Children Hospital University of Tripoli Tripoli Libya; ^17^ Third Department of Paediatrics, National and Kapodistrian University of Athens General University Hospital “Attikon” Athens Greece; ^18^ Pediatric Immuno‐Rheumatology Unit, Fondazione IRCCS ca’ Granda Ospedale Maggiore Policlinico Milan Italy; ^19^ Paediatric Rheumatology and Immunology, Vito Fazzi Hospital Lecce Italy; ^20^ Rheumatology, Allergology and Clinical Immunology, Department of System Medicine University of Rome “Tor Vergata” Rome Italy; ^21^ Department of Woman, Child and of General and Specialized Surgery University of Campania Luigi Vanvitelli Naples Italy; ^22^ Pediatric Rheumatology Unit, Department of Childhood and Developmental Medicine, Fatebenefratelli‐Sacco Hospital Milan Italy; ^23^ Department of Precision and Regenerative Medicine and Ionian Area (DiMePRe‐J) Policlinic Hospital University of Bari Bari Italy; ^24^ Department of Uvea and Ocular Inflammation Prabha Eye Clinic and Research Centre and Vittala International Institute of Ophthalmology Bengaluru Karnataka India; ^25^ Advanced Eye Centre Postgraduate Institute of Medical Education and Research Chandigarh India; ^26^ Department of Pediatrics, Giovanni XXIII Pediatric Hospital University of Bari Bari Italy; ^27^ Ophthalmology Department, Faculty of Medicine Cairo University Giza Egypt; ^28^ Ophthalmology Unit, Department of Medical Sciences, Surgery and Neuroscience University of Siena, Siena University Hospital [European Reference Network (ERN) for Rare Immunodeficiency, Autoinflammatory and Autoimmune Diseases (RITA) Center] Siena Italy

**Keywords:** adalimumab, methotrexate, monotherapy, noninfectious uveitis, paediatric ophthalmology

## Abstract

**Background:**

The study objective was to compare the effectiveness of adalimumab (ADA) in monotherapy and in combination with methotrexate (MTX) for paediatric noninfectious uveitis (NIU).

**Methods:**

Registry‐based observational study. Children receiving ADA for active uveitis were divided into the ADA monotherapy group (group 1) and the ADA plus MTX combination group (group 2).

**Results:**

Eighty four children were enrolled (146 eyes): 22 in group 1 (26.2%) and 62 in group 2 (73.8%). ADA effectiveness was complete in 48 children (57.1%), partial in 23 (27.4%) and absent in 4 (5.3%), without any differences across the groups (*p* = 0.89). Fewer relapses per 100 PY occurred after ADA treatment both in group 1 (280.0 vs. 23.0, *p* = 0.005) and in group 2 (297.9 vs. 86.0, *p* < 0.001). The final BCVA was similar between groups 1 and 2 [median 1.0 (IQR 0.3) and 1.0 (IQR 0.3), respectively, *p* = 0.55]. A statistically significant steroid‐sparing effect was observed in the entire cohort and in group 2 at the 6‐month (*p* = 0.01 and *p* = 0.01), 12‐month (*p* = 0.02 and *p* = 0.02), and last follow‐up (*p* = 0.045 and *p* = 0.045). The estimated ADA retention rate was 97.1% at 12 months, 87.7% at 24 months, and 82.6% at 36 months, without a statistically significant difference among the groups (*p* = 0.77).

**Conclusions:**

ADA monotherapy could be equally effective as its combination with MTX in both preventing uveitis relapses and preserving visual acuity in paediatric NIU, with comparable retention rates over 36 months of treatment. The steroid‐sparing effect of ADA monotherapy warrants further extensive evaluation to define its optimal placement in the therapeutic strategy for paediatric NIU.

## 1 Introduction

Biological therapies have revolutionised the treatment of paediatric non‐infectious uveitis (NIU), significantly improving both systemic and ocular outcomes of the disease. Adalimumab (ADA), a fully human monoclonal antibody targeting tumour necrosis factor (TNF), is the only biological treatment licensed for paediatric NIU in Europe and the United States. The efficacy and safety of ADA in treating NIU in children are well supported by data from randomised controlled trials, retrospective analyses from clinical registries, and case series studies [1, 2, 3, 4, 5, 6]. These trials proved that ADA, when combined with stable doses of methotrexate (MTX), effectively controls inflammation in children who do not respond to topical corticosteroids (CS) and first‐line disease‐modifying anti‐rheumatic drugs.

The therapeutic success of biological therapy in immune‐mediated diseases is closely linked to trough drug levels and drug concentrations at the target organs. In particular, failures with anti‐TNF agents have been associated with low systemic drug levels, which are influenced by factors such as body weight, pharmacokinetics, genetic profile, immunogenicity, and concomitant immunosuppressive treatment [7]. Immunogenicity can compromise therapeutic response by accelerating the clearance of monoclonal antibodies and potentially triggering severe infusion reactions. Although consensus is lacking, evidence indicates that NIU patients who develop anti‐ADA antibodies exhibit reduced trough ADA levels, leading to poorer clinical outcomes [8, 9]. Therefore, combination therapy with MTX is recommended to improve pharmacokinetics and reduce the risk of immunogenicity in patients undergoing ADA treatment. However, this approach is often limited in clinical practice due to adverse effects, such as hypertransaminasemia, and anticipatory or drug‐related nausea causing MTX intolerance in up to 40%–50% of children [10, 11].

In this context, given the limited availability of data on ADA monotherapy in paediatric NIU, we conducted a registry‐based analysis to identify potential differences in effectiveness outcomes of ADA monotherapy versus combination therapy with MTX in children with NIU.

## 2 Methods

This is a registry‐based observational cohort study leveraging data collected retrospectively and prospectively in the international AutoInflammatory Diseases Alliance (AIDA) Network registries dedicated to noninfectious uveitis and Behçet's disease [12, 13]. The registry records were screened for inclusion based on the following criteria: (1) diagnosis of NIU before the age of 18 years; (2) treatment with ADA started before the age of 18 years to control active uveitis refractory to local and/or systemic corticosteroids (CS) and/or conventional disease‐modifying anti‐rheumatic drugs (cDMARDs); (3) ADA given as monotherapy or in combination with MTX; (4) ADA treatment duration ≥ 6 months. Pseudonymized data extracted from the registries included demographic, clinical, and therapeutic information at the baseline (start of ADA treatment) and during the entire duration of ADA therapy.

Patients were divided into 2 study groups: ADA monotherapy (group 1) and ADA plus MTX combination therapy (group 2).

The study's primary objective was to compare the effectiveness of ADA monotherapy and combination therapy with MTX in controlling active ocular inflammation. With this respect, the study endpoint was a statistically significant difference between the study groups in terms of (1) global effectiveness of ADA treatment (complete, partial, or absent) and (2) change in the number of ocular relapses per 100 patients‐year before and after ADA treatment.

The secondary objectives of the study were to compare ADA monotherapy and combination therapy with MTX regarding (1) effectiveness in preserving visual acuity, (2) steroid‐sparing effect, (3) retention of ADA therapy over time, and (4) safety profile. With this regard, the respective study endpoints were defined as follows: a statistically significant difference across the study groups in (1) Best Corrected Visual Acuity (BCVA) at the last follow‐up, (2) percentage of children discontinuing systemic corticosteroids (CS) at 3‐, 6‐, 12‐ and last follow‐up, (3) drug retention rate of ADA at 12, 24 and 36 months and overall frequency of ADA discontinuation not due to remission, and (4) frequency of adverse events (AEs).

The global effectiveness of ADA was defined as complete if no relapses were observed after the start of treatment, partial in case of reduced frequency and/or severity of relapses, or absent if no improvement occurred. The treating physician attributed the anatomical classification of uveitis and the definition of ocular relapses according to the Standardisation of Uveitis Nomenclature (SUN) criteria [14]. Visual acuity, expressed as BCVA, was measured using age‐appropriate Snellen charts.

ADA was prescribed at the standard posology for paediatric uveitis treatment: 20 mg every other week for children weighing < 30 Kg or 40 mg every other week for children weighing ≥ 30 Kg. The MTX regimen was tailored according to the patient's body surface area and clinical requirements, as determined by the treating physician.

The study protocol adheres to the principles outlined in the Helsinki Declaration. Patients (or their parents/legal guardians) must provide written consent after receiving appropriate information to be eligible for the registry. The protocol of the international AIDA Network registries was approved by the Ethics Committee of Siena University Hospital (Ref. 14 951), and by the Ethics Committees of the participating investigator centres.

Statistical analysis was performed by using the JASP open‐source statistics package version 0.19.3.0. Descriptive statistics included counts and frequencies for categorical variables and mean and standard deviation (SD) or median and interquartile range (IQR) for continuous variables. The Shapiro–Wilk test was used to assess the normality distribution of data. Associations between categorical variables were analysed using contingency tables with the Chi‐Square test and Yates continuity correction. Differences in continuous data between independent groups were compared using the Mann–Whitney *U* test. Paired nominal data from subjects followed over time were compared using McNemar's test. Paired continuous data from subjects followed over time were compared using Wilcoxon's test. Time‐to‐event analysis was performed by the Kaplan–Meier method, with the event being ADA discontinuation for any reason except for remission. The survival curves were compared according to specific factors by the Log‐Rank test. The threshold for statistical significance was set to *p* < 0.05, and all *p* values were two‐sided.

## 3 Results

**FIGURE 1:**
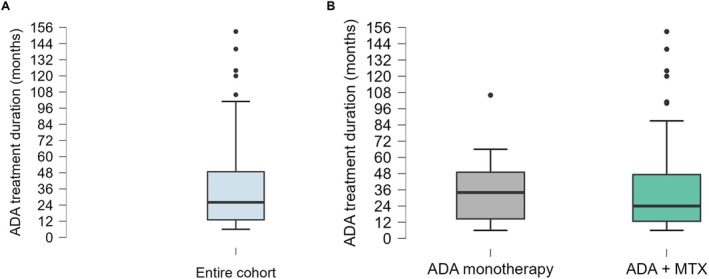
Visualisation of the distribution of adalimumab treatment duration in the entire cohort (A) and in the two study groups (B). List of abbreviations: ADA, adalimumab; MTX, methotrexate.

The study included 84 children (146 affected eyes) starting ADA treatment for active uveitis. Uveitis was idiopathic in 27 cases (32.1%), associated with an oculo‐specific clinical entity in 3 (3.4%) –HLA‐B27‐associated uveitis in 2 (2.2%), Vogt‐Koyanagi‐Harada syndrome in 1 (1.1%) – or with a systemic disease in 51 (60.7%) – JIA in 44 (52.4%), Behçet's disease in 5 (6.0%), inflammatory bowel disease and scleroderma in 1 case each (1.2%) (missing data in 3 children). ADA was employed as monotherapy (group 1) in 22 children (26.2%) and as combination therapy with MTX (group 2) in 62 children (73.8%). Among patients on combination therapy, MTX was started before or at the time of ADA introduction in 55 cases (88.7%), while it was added later during ADA treatment course in 4 (6.5%). The median (IQR) duration of ADA treatment was 26 (36) months (6–153) (Figure 1). Children were treated with ADA monotherapy for the following reasons: in 8 cases, MTX had been previously employed but proved ineffective and/or poorly tolerated; in 2 cases, ADA was prescribed off‐label to treat Behçet's disease; and in 12 cases, MTX had not been used before, and ADA monotherapy was prescribed according to the physician's and/or parents' preference.

Baseline characteristics of the study groups are summarised in Table 1.

**TABLE 1:** Baseline characteristics of the cohort stratified by study groups.

	Group 1 ADA monotherapy	Group 2 ADA plus MTX	*p*
*N* patients	22	62	—
*N* eyes	39	107	—
Male sex *N* (%)	7 (31.8)	22 (35.5)	0.96
Age at disease onset (years) Mean (SD) [min‐max]	8.7 ± 3.8 [3.5–15.5]	7.0 ± 3.6 [1.6–15.2]	0.09
Systemic disease association *N* (%)	13 (59.1)	38 (61.3)	0.54
ANA *N* patients (%)	7 (31.8)	28 (45.2)	0.37
Granulomatous uveitis *N* eyes (%)	6 (19.4)	14 (16.7)	0.55
Ocular complications *N* eyes (%)^a^	19 (48.7)	53 (49.5)	0.93
Uveitis anatomical classification			
Anterior *N* eyes (%)	23 (59.0)	66 (62.9)	0.07
Posterior *N* eyes (%)	2 (5.1)	0 (0.0)	
Panuveitis *N* eyes (%)	7 (17.9)	27 (25.7)	
Intermediate *N* eyes (%)	7 (17.9)	12 (11.4)	
Uveitis course			
Acute *N* eyes (%)	8 (22.2)	13 (14.3)	0.43
Recurrent *N* eyes (%)	11 (30.6)	37 (40.7)	
Chronic *N* eyes (%)	17 (47.2)	41 (45.1)	
Past treatments			
Previous CS drops *N* (%)	17 (77.3)	38 (62.3)	0.10
Previous regional CS *N* (%)	1 (4.5)	3 (4.8)	0.51
Previous dilator or cycloplegic *N* (%)	15 (68.2)	41 (66.1)	0.45
Previous systemic CS *N* (%)	8 (36.4)	13 (21.0)	0.23
Previous cDMARDs *N* (%)	8 (36.4)	50 (80.6)	0.39
Previous MTX *N* (%)	8 (36.4)	50 (80.6)	**< 0.001**
Previous bDMARDs *N* (%)	3 (13.6)	14 (22.6)	0.56
Age at ADA start (years) Mean (SD) [min‐max]	10.1 ± 3.5 [4.0–16.0]	8.8 ± 3.8 [2.0–18.0]	0.17
Disease duration at ADA start (months) Median (IQR) [min‐max]	11.0 (26.8) [3.0–108.0)	17.5 (31.5) [0.0–131.0]	0.75
Active extraocular symptoms at ADA start *N* (%)	4 (18.2)	14 (22.6)	1.00
BCVA at ADA start Median (IQR) [min‐max]	1.0 (0.00) [1.0–1.0]	0.95 (0.30) [0.03–1.00]	—
ADA treatment duration (months) Median (IQR) [min‐max]	34.0 (34.5) [6.0–106.0]	24.0 (35.0) [6.0–153.0]	0.88
ADA biosimilar *N* (%)	12 (54.5)	27 (43.5)	0.57
Biologic naive *N* (%)	20 (90.9)	54 (87.1)	0.80
MTX starting dosage mg/mq/week Median (IQR) [min‐max]	—	15.0 (5.0) [7.5–25.0]	—
CS at ADA start *N* (%)	4 (18.2)	19 (30.6)	0.58

Abbreviations: ADA, adalimumab; ANA, antinuclear antibodies; BCVA, best corrected visual acuity; bDMARD, biological disease‐modifying anti‐rheumatic drug; cDMARD, conventional disease‐modifying anti‐rheumatic drug; CS, corticosteroids; IQR, interquartile range; MTX, methotrexate; SD, standard deviation.

^a^ Ocular complications included the following: Macular edema in 28 eyes (19.2%), cataract in 28 (19.2%), posterior synechiae in 22 (15.1%), open‐angle glaucoma in 10 (6.8%), band keratopathy in 18 (12.3%), peripheral anterior synechiae in 6 (4.1%), steroid‐induced cataract in 7 (4.8%), steroid‐induced ocular hypertension in 3 (2.0%), choroidal neovascularization in 3 (2.0%), retinal pigment epithelial alterations in 3 (2.0%), retinoschisis in 2 (1.4%), retinal fibrosis in 3 (2.0%), retinal ischemia in 2 (1.4%), retinal edema in 2 (1.4%), other complications in 1 (0.7%) each.

**FIGURE 2:**
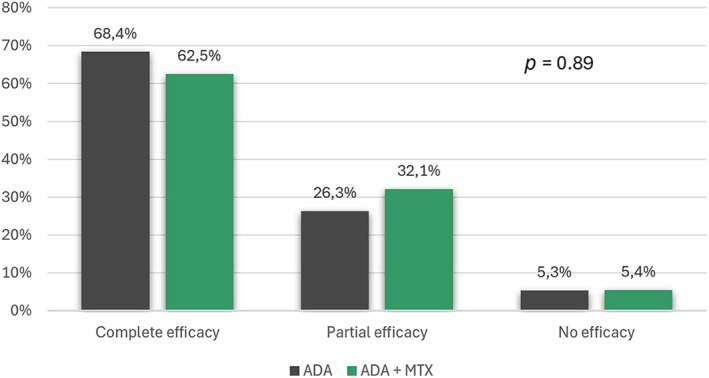
Percentage of children with complete, partial, or no effectiveness of adalimumab treatment across the study groups. No statistically significant differences were found between the groups (*p* = 0.89). List of abbreviations: ADA, adalimumab; MTX, methotrexate.

Global effectiveness of ADA therapy was defined as complete in 48 children (57.1%), partial in 23 (27.4%), and absent in 4 (5.3%) (data not available in 9 children). No statistically significant difference was found across the study groups regarding ADA global effectiveness, which was complete in 13 (68.4%) children in group 1 and 35 (62.5%) in group 2, partial in 5 (26.3%) children in group 1 and 18 (32.1%) in group 2, and absent in 1 (5.3%) child in group 1 and 3 (5.4%) in group 2 (*p* = 0.89) (Figure 2).

In the whole cohort, the number of relapses per 100 patients before and after starting ADA were 293.5 and 67.4, respectively (*p* < 0.001). When stratifying the cohort by study groups, fewer relapses per 100 PY occurred after ADA treatment both in group 1 (280.0 vs. 23.0, *p* = 0.005) and in group 2 (297.9 vs. 86.0, *p* < 0.001).

The median BCVA in the whole cohort was 1.0 (IQR 0.3) [range 0.025–1.0] at the baseline and 1.0 (IQR 0.3) [range 0.025–1.0] at the end of the follow‐up (*p* = 0.40). No difference was found in the median BCVA at the end of the follow‐up across the study groups: 1.0 (IQR 0.3) [0.025–1.0] in group 1 and 1.0 (IQR 0.3) [0.025–1.0] in group 2, with *p* = 0.55.

**FIGURE 3:**
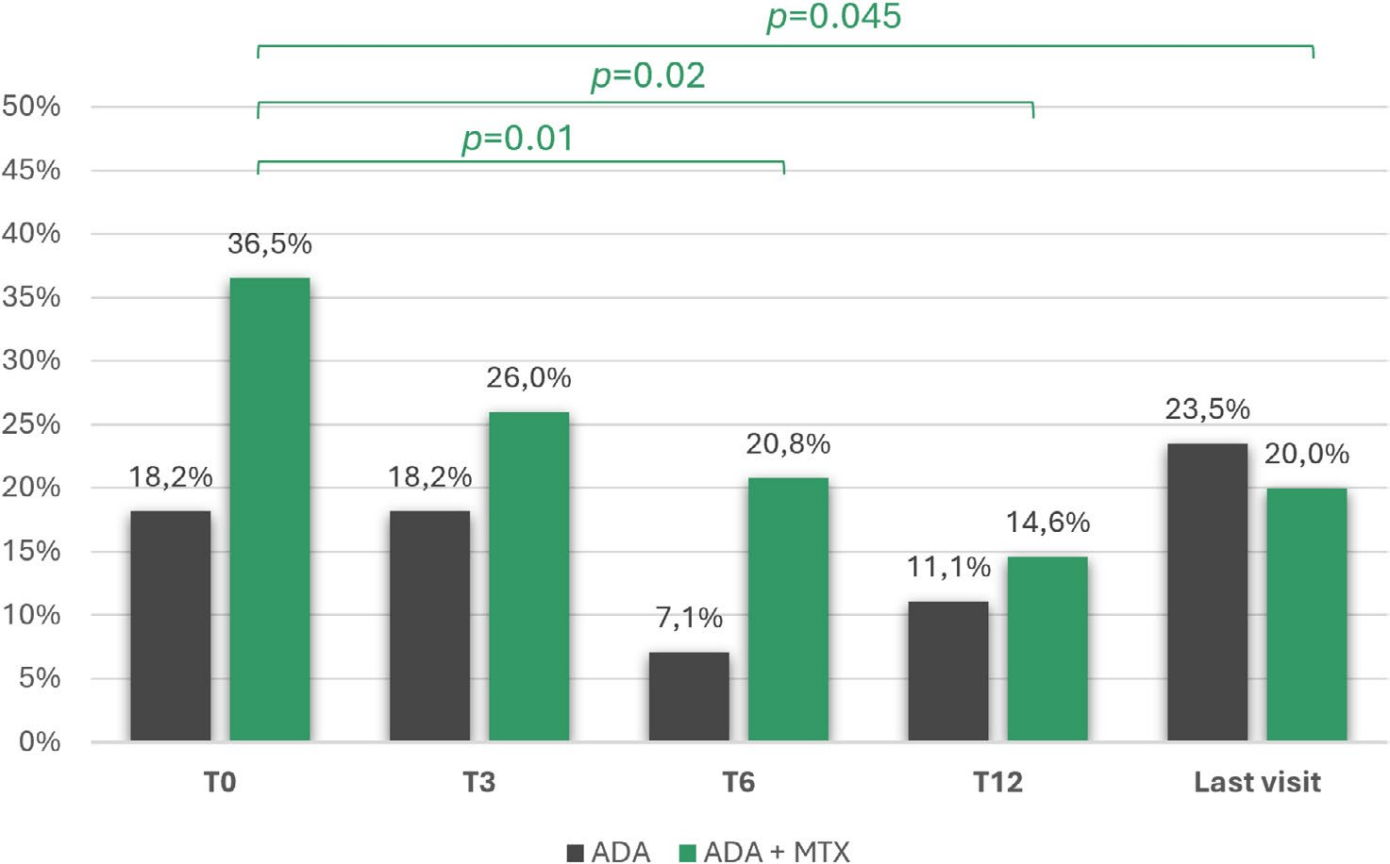
Percentage of children receiving corticosteroids at different timepoints after the start of adalimumab. Percentages are calculated on the total number of children with available information at each timepoint in the two groups. Statistical significance has been evaluated between the baseline and each timepoint. List of abbreviations: ADA, adalimumab; MTX, methotrexate.

Systemic CS were taken by 23 (33.8%) children at the baseline, 17 (26.2%) after three months (*p* = 0.45), 11 (17.7%) after six months (*p* = 0.01), 7 (14.0%) after 12 months (*p* = 0.02), and 15 (20.8%) at the last follow‐up available (*p* = 0.045). When stratifying the cohort by study groups, the statistical significance was maintained only in the combination therapy group at the 6‐month (*p* = 0.01), 12‐month (*p* = 0.02), and last follow‐up (*p* = 0.045) (Figure 3).

**FIGURE 4:**
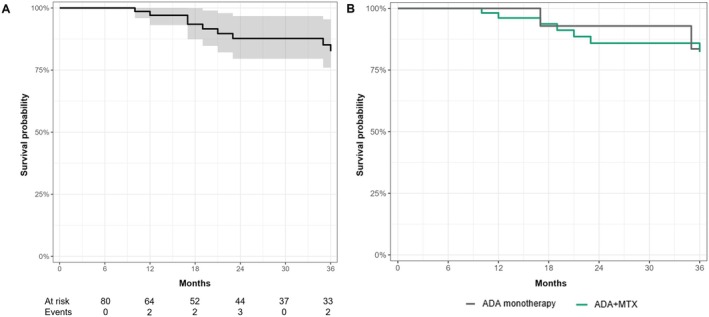
Adalimumab survival curve for the whole cohort (A) and stratified by study groups (B). The Kaplan–Meier curves display survivorship up to 36 months. Patients with shorter follow‐up durations are censored at their last observation. The number of patients at risk of discontinuation and the number of patients discontinuing adalimumab – for reasons other than remission – are reported at each timepoint. List of abbreviations: ADA, adalimumab; MTX, methotrexate.

The estimated ADA retention rate in the entire cohort was 97.1% at 12 months, 87.7% at 24 months, and 82.6% at 36 months (Figure 4A). No statistically significant differences were found in ADA survival across the two study groups (*p* = 0.77) (Figure 4B). Adalimumab was discontinued by 17 patients (20.2%) overall: 4 (23.5%) in remission, 3 (17.6%) reporting AEs, 4 (23.5%) reporting lack of effectiveness on the ocular and/or extraocular disease, 3 (17.6%) reporting loss of effectiveness on the ocular and/or extraocular disease, and 3 (17.6%) for other medical or non‐medical reasons. Discontinuation of ADA not due to remission was reported in 3 children (13.6%) in group 1 and 10 (16.1%) in group 2 (*p* = 1.00).

Adverse events (AEs) were reported in 8 children (9.5%): pain at the injection site, psoriasiform skin rash, and recurrent umbilical infection were reported in one patient; migraine and mood changes in another one; cataract, elevation of liver enzymes, HSV infection, lymph node swelling, diagnosis of multiple sclerosis, and vulvovaginitis were reported in one patient each. AEs were reported in 3 children (14.3%) in group 1 and 5 (8.1%) in group 2 (*p* = 0.72).

## 4 Discussion

This study provides real‐life data about ADA effectiveness in children and adolescents with active NIU undergoing monotherapy or combination therapy with MTX. The treating physician judged the ADA treatment course as effective in most patients, regardless of the concomitant use of MTX. Also, ADA could prevent uveitis relapses over time and preserve visual acuity, both in monotherapy and in combination with MTX, which resulted in optimal long‐term survival. Furthermore, a low frequency of AEs has been reported, supporting the safe use of these drugs in young patients.

A limited number of studies in the paediatric and adult populations have evaluated the use of ADA in monotherapy to treat NIU. Al‐Julandani and colleagues investigated ADA effectiveness after discontinuing cDMARDs in 28 children with anterior or intermediate uveitis. More than 80% of them met the primary outcome of the study, having less than a 2‐step worsening in uveitis according to the SUN and the Nussenblatt scores [14, 15], with no addition of other cDMARDs during follow‐up. Furthermore, they estimated an 81.3% probability of maintaining control of uveitis on ADA monotherapy at 12 months [16]. As for children affected by non‐anterior uveitis, a previous study on 21 children from the AIDA Network registries found a similar proportion of subjects on combination therapy with ADA plus cDMARDs (including MTX in 47.6% of cases) in the group of complete and partial responders [17]. Another observational study conducted in the adult population questioned the real contribution of cDMARDs to ADA efficacy and drug retention rate in subjects with uveitis associated with systemic immune‐mediated diseases [18]. By comparing 29 patients on ADA monotherapy and 29 on combination therapy, who were homogeneous in terms of baseline ocular inflammation, they observed no differences in terms of ocular disease activity control and steroid‐sparing effect over 12 months.

Further evidence derives from studies on children with JIA, which made up half of the diagnoses in the present cohort. Data from the German Biologics in Paediatric Rheumatology (BiKeR) registry on 584 children with non‐systemic JIA on ADA therapy showed no differences between monotherapy and MTX combination in multiple outcomes, including last follow‐up rates for JIA American College of Rheumatology (ACR) 30/50/70/90 response, Juvenile Arthritis Disease Activity Score (JADAS)‐based minimal disease activity and remission, and ACR inactive disease, as well as a similar rates of AEs and severe AEs. In the same cohort, no uveitis flares were reported at 12 and 24 months, and the last follow‐up in 78%, 86% and 83% of patients on ADA monotherapy and in 84%, 80%, and 80% of patients on combination therapy, respectively [19]. In addition, data from the long‐term extension period of a phase III trial of ADA in polyarticular JIA confirmed that ADA with and without background MTX is effective in long‐term treatment with similar therapeutic outcomes and safety profiles. Nevertheless, the comparison between observed and non‐responder imputation analyses of JIA ACR and JADAS27 responses suggests a higher drop‐out rate in the monotherapy group [20].

Our results contrast with some observations suggesting that the concomitant use of MTX may lower the probability of developing neutralising anti‐ADA antibodies and, consequently, the risk of enhanced ADA clearance and therapeutic failure. In a small prospective study on JIA‐associated uveitis, Skrabl‐Baumgartner and colleagues effectively demonstrated a link between the presence of permanent anti‐ADA antibodies, reduced ADA trough levels, loss of therapeutic response and ADA monotherapy, thus suggesting that cDMARDs should be maintained even after remission is achieved with combination therapy [21]. Comparable results were observed in a Finnish study of 31 children with JIA‐related uveitis and in a retrospective cohort study involving 42 adults with NIU receiving ADA with or without antimetabolites [9, 22].

Although the hypothesis that MTX directly preserves ADA efficacy over time is appealing, the data on this aspect remain controversial. In a Spanish prospective study on 25 subjects with NIU, concomitant cDMARD therapy did not show any protective effect from anti‐ADA antibody development [8]. Similarly, Rusche et al. failed to demonstrate a correlation between MTX use and the detection of anti‐ADA antibodies in a small cohort of 18 children with NIU [23]. More recently, a retrospective, observational pilot study identified a therapeutic range for ADA trough levels in the treatment of paediatric NIU, ranging from 9.6 to 13 μg/mL, which may result in an optimal clinical effect. Notably, ADA trough levels were comparable in the monotherapy and MTX combination therapy groups. Also, a similar percentage of children reached a complete response in the two groups (73% with combination therapy and 66% with monotherapy) mirroring the results of the present study [24]. It must be underlined that, in this cohort, approximately 36% of children on ADA monotherapy had previously been treated with MTX; thus, a possible tail effect of MTX cannot be excluded. Additionally, the choice of ADA monotherapy instead of combination therapy with MTX was driven by various factors—such as prior MTX ineffectiveness or poor tolerance, off‐label indication, or a general preference by the physician and/or the family—which makes it difficult to rule out that more severe cases may have been started on dual treatment in anticipation of potential treatment failure. Given these limitations, along with the lack of data on anti‐ADA antibody development and ADA trough levels in this cohort, conclusive considerations cannot be drawn. However, only 3 out of 84 patients (1 on monotherapy and 2 on combination therapy) discontinued ADA due to secondary ineffectiveness, indirectly suggesting that ADA levels were maintained within the therapeutic range for most children. This is consistent with the high drug retention rates estimated for children on both ADA monotherapy and combination therapy.

The goal of achieving steroid‐free remission promptly is considered crucial in the paediatric setting to avoid ocular and systemic complications and prevent structural damage to the eye and growth retardation [25]. Most children from this cohort did not require systemic CS at the baseline, and the percentage of children taking CS further decreased during ADA treatment, confirming its favourable steroid‐sparing effectiveness. Notably, children on MTX combination therapy exhibited a more consistent pattern of CS discontinuation compared with the monotherapy group, although low numbers and missing information may have impacted the statistical significance in the monotherapy group. In this regard, the study by Bitossi and colleagues found that the use of cDMARDs did not influence the need for CS in adult patients with uveitis associated with systemic immune‐mediated diseases receiving ADA treatment [18]. Given the relatively small size of the present cohort and the conflicting evidence available, more extensive research is warranted to evaluate the steroid‐sparing effectiveness of ADA monotherapy compared to combination therapy, aiming to elucidate the optimal use and timing of ADA monotherapy in paediatric patients with NIU. Specifically, it is crucial to determine whether combination therapy should remain the preferred induction treatment for children with CS dependence, while ADA monotherapy may be more appropriate for maintenance therapy after achieving therapeutic effects or in cases of MTX intolerance.

In conclusion, ADA monotherapy may be as effective as combination therapy with MTX in preventing uveitis relapses and preserving visual acuity in children with NIU, with an acceptable safety profile. Additionally, both therapeutic regimens exhibit an excellent retention rate over 36 months, with very few children discontinuing ADA due to secondary ineffectiveness, thereby questioning the substantial clinical impact of anti‐ADA antibody development. Nonetheless, further extensive evaluation is needed to fully understand the steroid‐sparing effect of ADA monotherapy and its optimal placement in the treatment strategy for children with NIU. Based on the findings of this study, ADA monotherapy could be a viable therapeutic option for children with active uveitis in instances of MTX intolerance or contraindication.

## Conflicts of Interest

The authors declare no conflicts of interest.

## Data Availability

The datasets generated during and/or analysed during the current study are available from the corresponding author on a reasonable request.
